# Prime editing in mammals: From promise to practicalities

**DOI:** 10.1016/j.omtn.2025.102719

**Published:** 2025-09-20

**Authors:** Imogen R. Brooks, Carina Graham, Aidin Kazemizadeh, Joanna Jacków-Malinowska

**Affiliations:** 1 St. John’s Institute of Dermatology & KHP Centre for Translational Medicine, King’s College London, London SE1 9RT, UK

**Keywords:** MT: RNA/DNA Editing, gene editing, genetics, prime editing, off-target effects, high-throughput sequencing, genome engineering

## Abstract

Prime editing (PE) is a recent advancement in CRISPR-Cas9 technology that involves the fusion of a reverse transcriptase (RT) to a Cas9 nickase (nCas9). This fusion protein is complexed with a prime editing guide RNA (pegRNA), which includes both a spacer sequence for Cas9 targeting and a template used by the RT to install desired edits into the genome. This system can generate small-scale insertions, deletions, and substitutions while elegantly bypassing double-stranded break formation, reducing the risk of unwanted indels and off-target editing. However, there is high variability in editing reported by different studies using different methodologies and disease models. In this review, we systematically examine how PE performs in different models and how different approaches improve or hinder PE efficiency. Furthermore, as assessing DNA editing efficiency is a time-consuming process, we discuss reporter assays used to detect editing events and select for edited cells. Finally, we examine the detected downstream effects of PE and investigate potential explanations for variability between models. Taken together, this review provides a valuable insight for researchers as to how PE may perform in their chosen cellular and animal models and how to effectively analyze and troubleshoot their PE experiments.

## Graphical abstract

**Figure:**
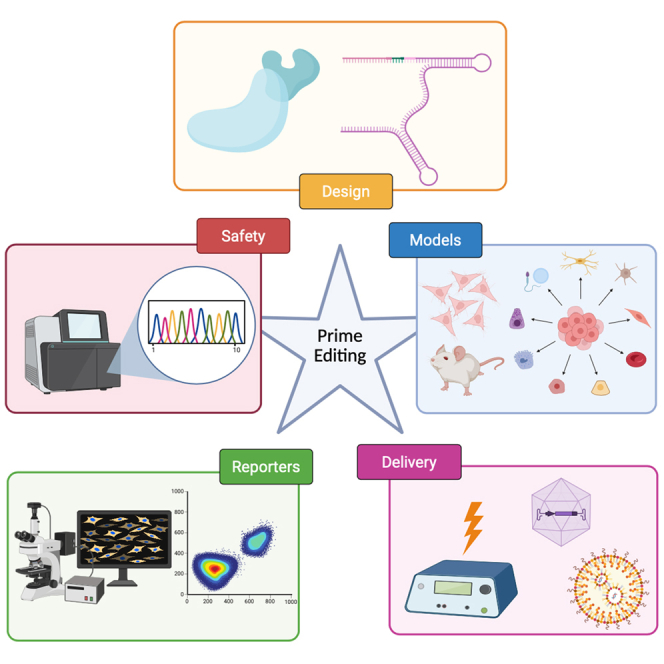

## Teaser

Jacków-Malinowska and colleagues discuss the exciting field of prime editing, a gene editing approach based on CRISPR-Cas technologies. However, it can be challenging to bring bench side functionally. This review discusses strategies published in the literature, how prime editing efficiency can be measured, and what safety considerations are relevant.

## Introduction

Prime editing (PE) is a versatile, cutting-edge field of gene editing,^1^ but the excitement around its potential is often curbed by challenges to apply it at the bench. PE is a gene editing approached based on the original premise of CRISPR/Cas9 editing. Typically, in CRISPR/Cas9, a Cas9 protein is expressed within a cell along with a guide RNA (gRNA), which directs the Cas9 to a specific sequence of homology proximal to a protospacer-adjacent motif (PAM) site. The Cas9 nuclease cleaves the DNA, resulting in a double-stranded break (DSB). The cell’s endemic systems then initiate repair of the DSB, either through nonhomologous end joining (NHEJ) or through homology-directed repair (HDR). By introducing a donor template for the HDR mechanism, an edit can be encoded at the specific locus. However, in many cell lineages, NHEJ is the preferred repair pathway, resulting in deleterious indels (insertions and deletions).^2^

PE bypasses both NHEJ and HDR by avoiding DSBs, using a Cas9 nickase (nCas9), which is catalytically impaired to only induce a single-strand break (SSB), which is fused to a reverse transcriptase (RT) to encode edits.^1^ Using a PE gRNA (pegRNA), the nCas9 induces an SSB at the desired locus. Then, the RT encodes the edit on the nicked strand via a reverse transcription template (RTT) included in the pegRNA. Through the endogenous repair mechanisms, the edit on the nicked strand is copied to the complementary strand. This reduces both the risk of indels and the number of elements that must be transduced into a cell, reducing variability.

**Figure 1:**
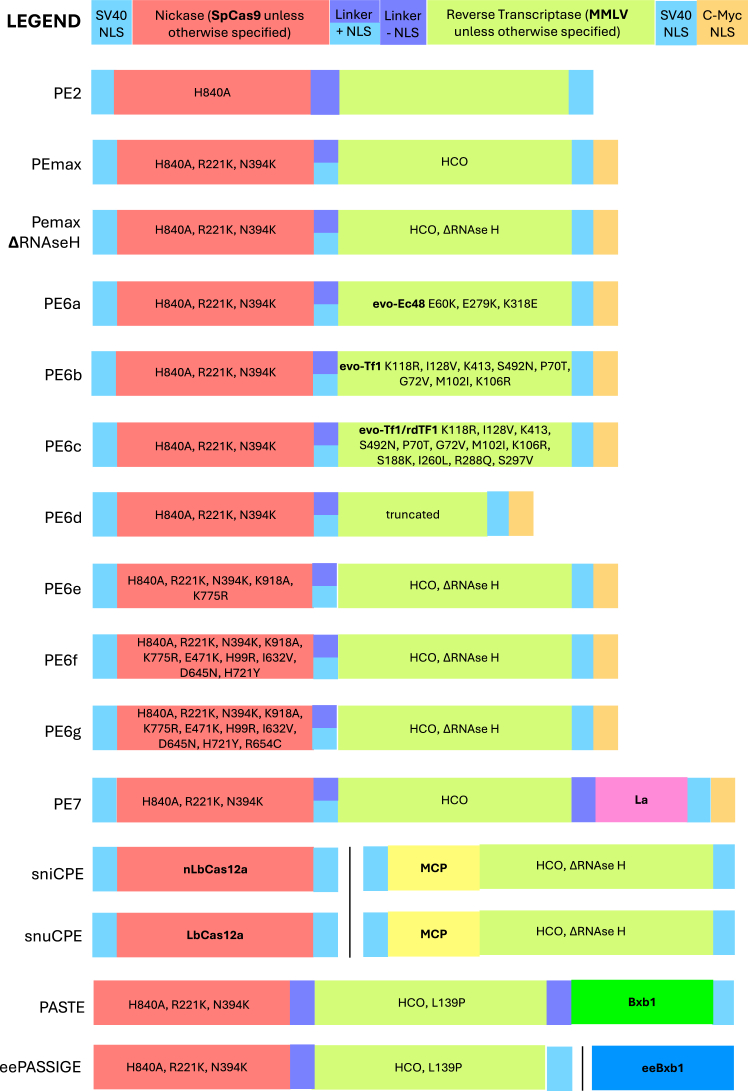
Schematic of engineered and evolved PE architectures Common features are color-coded according to the legend (top). Amino acid changes, RNAse H deletion, codon optimization, and alternative proteins indicated in each feature’s box. Protein names in bold text. NLS, nuclear localization signal; SpCas9, *S. pyogenes* Cas9; MMLV, Moloney murine leukemia virus; HCO, human codon optimized; MCP, MS2 coat protein. sniCPE, snuCPE, and PASSIGE function with two independent constructs, with the split between them indicated by a thin vertical black line.

Several PE proteins have been published, although PE2 (Figure 1) and PEmax are the main PE tools currently in use, due to the recency of other published models. PE1 and PE2 were first developed from a wild-type M-MLV RT and a modified M-MLV RT, respectively, with PE2 outperforming PE1.^1^ PEmax was further developed from PE2 by codon optimizing the RT, adding two additional nuclear localization signals (NLSs), and introducing mutations to the SpCas9, which improve the nuclease activity.^3^ PEmax performs better than PE2 at many genomic loci and performs equally at other genomic loci. As such, PEmax is the recommended protein to use in PE applications.^4^ More recently, other PE proteins have been published such as the PE6 suite and PE7 (Figure 1),^5^^,^^6^ which will be discussed further below. Beyond the PE protein itself, efficiency of PE can be improved with additional systems, labeled PE3, PE4, and PE5.^3^ PE3 adds an additional nicking single-guide RNA (sgRNA) (ngRNA) that targets the alternate strand, increasing editing efficiency by causing mismatch repair to favor the edited strand, but results in a risk of DSBs and higher indels.^1^ PE4 transiently inhibits MMR with a dominant negative MLH1 protein, ensuring MMR does not occur until after flap integration.^3^ PE5 is the combination of both strategies. With each addition, further optimization is required, and an increase in editing efficiency is not guaranteed.

In this review, we will explore the steps of optimizing PE experiments for mammalian applications. We will take the reader through the process from efficiency to optimization, model-specific considerations, quantification of successful editing events at the locus of interest, and finally to detection of off-target effects (OTEs). We consider how those practicalities impact PE experiments and propose or reiterate recommendations for PE experimental design.

## Efficiency of editing

The efficiency of PE poses one of the most significant hurdles in the experimental approach. Low editing efficiency is more difficult to detect and requires higher sensitivity and therefore usually more expensive detection approaches, such as next-generation sequencing (NGS).^7^ Isolating edited cells to establish isogenic cell lines also poses a challenge. Many elements are at play: the model being used, the PE machinery being used, whether an engineered pegRNA (epegRNA) or a non-engineered pegRNA is used, in what form the PE is delivered, the transfection mechanism, and whether to use selection to enrich for successful transfection. In this section, we will consider the literature and offer recommendations for optimizing the efficiency of PE experiments.

### PE protein

Of the papers read for this review, published in the last 18 months, most papers have been published using the PE2 system, although there is an increase toward the use of PEmax (Table 1). This is likely due to the time-consuming and expensive nature of PE studies, especially if viral vectors or mRNA must be synthesized and optimized. It is anticipated that more and more studies will favor the PEmax system, as studies done both by the original authors and other research groups have found that the PEmax protein matches or surpasses the efficacy of PE2.^3^^,^^25^

**Table 1:** A summary of key prime editing results discussed in this review

References	Type of edit (gene)	Model	Max efficiency	PE system	Delivery (transfection)	Enrichment	Off-target edits (method)	Notes
Liang et al.^8^	CGCCCCG> ATGGTAT (*HEK2*)	HEK293	0.4075	Cas12a PE2, split nickase	plasmid (lipofection)	None	<0.3% (targeted amplicon NGS)	circular epegRNA
Ponnienselvan et al.^9^	G>T (*FANCF*)	human fibroblasts	∼22%	PEMax	mRNA (electroporation)	None	not performed	cold shock of cells enhanced editing
Oliveros et al.^10^	T>C (*TRAPPC910*)	HEK293	NR	PE2	plasmid (lipofection)	Single-cell cloning	not performed	repeat transfection and limited dilution cloning used
Anzalone et al.^11^	deletion, replacement, integration, inversion (up to 40 kb inversion)	HEK293T; HeLa and Huh7	90 bp replacement 20–46%; >5 kb plasmid integration 12–17%	twinPE (paired pegRNAs with complementary flaps) ± recombinase	plasmid	None	targeted amplicon sequencing	
Peretto et al.^12^	C>T (*SBDS*)	HEK293	NR	PE2	plasmid (lipofection)	None	not performed	minigene model
Tao et al.^13^	large fragment deletions, multiplex edits	HEK293T; K562; HeLa, B16	deletion up to 64.4% (1,522 bp); examples: 36.7% (400 bp), 31.5% (861 bp); replacement up to 60.8% at HEK3	Bi-PE (paired pegRNAs; inward nick orientation, Type II design)	plasmid transfection	None	targeted amplicon NGS; indels lower than PE3 (e.g., 0.35% vs. 4.7–8.2% at HEK3)	Bi-PE-3 generally outperforms Bi-PE-2 and PE3; worked across multiple loci and cell types
Choi et al.^14^	deletions up to 10 kb (eGFP, genomic loci)	HEK293T	1%–30% (depending on locus and size)	PE2 with paired pegRNAs (two guides on opposite strands that define the boundaries of the programmed deletion	plasmid	Extended expression improved efficiency	none	higher precision than Cas9/sgRNA; concurrent deletion + short insertions possible
Zhuang et al.^15^	base substitutions, insertions (e.g., 24 bp), and deletions with paired pegRNAs (PAM-in)	HEK293T; HCT116 (MMR-deficient)	insertion (24 bp at HEK site3) 32.6%; generally higher than PE2/PE3; improved purity vs. PE3	HOPE, paired pegRNAs (with PAM sites oriented inward toward the edit)	plasmid transfection	None	high-sensitivity targeted amplicon sequencing with UMIs: very low off-target; similar to PE3	design tips: PBS Tm ∼35°C–40°C; RT downstream length ∼7–15 nt
Jiang et al.^16^	large deletions (1–10 kb) with insertions (≤60 bp)	HEK293T; mouse Fah−/− model	17.7% deletion + insertion at HEK3	PE-Cas9 (nuclease fused to RT) + pegF/pegR, PE-Cas9-based deletion and repair	plasmid	None	none	corrected 1.38 kb insertion *in vivo*; restored FAH expression
Kweon et al.^17^	targeted translocation/inversion	HEK293 cells; A549 cells	0.04	PE2	plasmid (lipofection)	None	targeted deep sequencing; multiplex PCR (to identify unwanted translocation)	paired pegRNAs
Wang et al.^18^	donor-free large insertions (20 bp to ∼1 kb) with deletion between nicks	HEK293T-EGFP (primary); also, endogenous loci (*FANCF, HEK3/4, VEGFA, LSP1, PSEN1*); K562, Huh-7, N2a; non-dividing RPE	accurate insertion up to 43.7% (150 bp); 42.7% (101 bp); larger cargo: 0.38% (458 bp) and ∼0.002%–0.003% (600–1,085 bp)	GRAND(paired pegRNAs with complementary RTTs)	plasmid transfection; best with calcium phosphate/PEI (recommendation)	None (drug selection used only to verify BSD function)	predicted Cas9 off-targets showed no indels by amplicon sequencing	works in non-dividing cells; avoid RTT microhomology; 50–60 nt complementary overlap worked best
Xiong et al.^19^	upstream-to-nick edits; replacement (37 bp), deletion (37 bp), and simultaneous both-sides edits	HEK293T	replacement 33.7%; deletion 18.8%; simultaneous both-sides 10.7%; up to122.1× fold-improvement vs. PE2 in a large-fragment example	EXPERT	plasmid transfection	None	low indel rates (e.g., 0.28% at HEK4_2) and no genome-wide off-target increase reported	enables upstream editing beyond pegRNA nick; simultaneous upstream + downstream edits
She et al.^20^	T>C (*Rpe65*)	mouse model	0.16	PE3	AAV (subretinal injection)	None	none detected (NovaSeq amplicon sequencing)	PE architecture split across two AAV vectors
Davis et al.^21^	C>T (*Dnmt1*)	mouse model	46% (liver), 42% (brain), 11% (heart)	PE3	AAV (intracerebroventricular injection)	FACS	none detected (CIRCLE-seq)	editor split across two AAVs
Brooks et al.^22^	C>T (*Pah*)	mouse model	0.52	PEMax	AAV (retro-orbital injection)	None	none detected (ONE-seq)	
Wang et al.^23^	1-bp insertions or 2-bp deletions at *DMD* exon 51	human myogenic cells	50%–60%	PE2 and PE3	Adenovirus	None	6%–19% (MiSeq amplicon sequencing)	
Xia et al.^24^	*Lhgcr* c.1485G>A (W495X)	stem Leydig cells in mice	0.2342	PEMax	lentiviral transduction	None	none detected (targeted deep sequencing)	
Godbout et al.^25^	T>C (*RYR1*)	human myoblasts	0.59	PE2	mRNA (electroporation)	None	not performed	3 repeated electroporations; silent mutation induced in target codon
Binder et al.^26^	G>T (*ACTB*)	iPSC	NR	PEMax	plasmid (lipofection)	FACS	0% (Oxford nanopore sequencing)	
Li et al.^27^	G>A (*CFTR*)	iPSC-derived airway epithelial cells	0.024	PE2	adenovirus (in culture media)	None	not performed	
Fiumara et al.^28^	not specified (KO *B2M*)	K-562	0.9	PE3	mRNA (electroporation)	None	“alteration of the exome mutational landscape” (whole exon sequencing)	
Li et al.^29^	T>A (*HBB*)	mouse model	0.42	PE5max	adenovirus (intravenous)	None	none detected (CIRLCE-Seq)	
Nguyen et al.^7^	prime editing (e.g., insertions or deletions via pegRNA-guided modification)	baby Syrian hamster kidney cells (BHK-21)	NR	PE2	plasmid (TransIT-2020)	Antibiotic resistance, FACS	quantified via Illumina MiSeq NGS for high-resolution analysis	
Frisch et al.^30^	C>A (*RNF2*)	hPSCs	∼80%	PE2	plasmid (lipofection)	PINE-TREE reporter	none detected (Sanger sequencing)	
Simon et al.^31^	1 bp deletion (*RUNX1*)	HEK293	0.85	PE2	plasmid (lipofection)	PEAR reporter	none detected (GUIDE-seq)	
Wimmer et al.^32^	delA (*ABCA4*)	HEK293	0.92	PE3	plasmid (polyethylenimine transfection)	BRET reporter	not performed	
Kleinboehl et al.^33^	38-bp insertion (*CCR5*)	K-562	∼12%	PEmax	plasmid (electroporation)	V-GEAR reporter	not performed	
Li et al.^34^	CTT insertion	K-562	0.94	PE2	plasmid	FACS	not performed	

NR stands for “not recorded”.

There have been a few studies aiming to further optimize the PE protein. One of the major challenges with PE is the size of the PE protein, which poses a challenge for packaging in AAV vectors,^35^ transfection efficiency,^6^^,^^36^ and mRNA synthesis.^37^ Replacing the Cas9 nuclease in PE2 with Cas12 reduces the size of the PE protein and would facilitate editing in T-rich regions of the genome; however, this comes at the expense of reduced editing efficiency and specificity.^8^^,^^38^^,^^39^ Further work is needed to compare this approach to Cas9-dependent editing and to elucidate the optimal conditions to favor a Cas12 approach over a Cas9 approach.

In the last year, Doman et al. have published a suite of new PEs—PE6a-g.^6^ In brief, through phage-assisted evolution, compact PE proteins up to 810 base pairs (bp) smaller than PEmax were developed, with different applications dependent on cell type and edit type. For example, PE6a is preferential when looking for the smallest possible prime editor, whereas viral delivery approaches are better suited for PE6d and PE6d. Outside of the originating lab, not much has been published with these prime editing proteins yet, but they offer new flexibility for optimizing prime editing delivery.^6^

Recently, another PE protein has been published—PE7, which incorporates a La protein into the PEmax architecture, (Figure 1) enhancing RNA binding to the pegRNA to increase the editing efficiency on most target loci tested, by a median of 2.3-fold improvement.^5^ Trialling this approach could be valuable for difficult-to-correct loci. Alternatively, increasing the DNA binding affinity to the single-stranded DNA flap can be achieved by incorporating the RAD51 domain into the PE as seen in HyPE2, which improves efficiency over the PE2 protein;^40^ however, this has not yet been tested in the PEMax or PE6 architectures.

Additionally, it is further possible to extend the functionality of the PE by using alternative Cas9 proteins to the canonical SpCas9 protein, introducing further PAM flexibility. This has been demonstrated by using modified SpCas9 proteins such as PE2-SpG, which targets an NGH PAM, and the PE2-SPRY, which is PAMless, albeit with reduced activity.^41^ Alternatively, one could extend prime editing functionality by replacing the SpCas9 with an engineered Cas9 from *Francisella novicida*, EnFnCas9. This Cas9 has shown enhanced specificity for the target site while using a flexible NRG/NGR PAM site, with far fewer off-target effects detected with Digenome-seq relative to canonical SpCas9.^42^ This could further expand the functionality of PE proteins by reducing the PAM-related limitations on that exist with the current SpCas9 protein. Taken together, depending on precise experimental needs, it may be worth considering alternative prime editing protein modifications depending on the specific target of interest.

### pegRNA motif

There are three crucial components within a pegRNA which require optimization: the spacer, the RTT, and the primer binding site (PBS).^43^ Recommendations for basic pegRNA design have remained standard: typically, the spacer is ideally located as close as possible to the desired locus so that the nicking site is directly 5′ of the target locus. The PBS is optimally between 10 and 15 bp in length. RTT length can vary dependent on the desired edit. For smaller edits, an RTT with minimum seven bases of complementarity after the edit is desirable, but for larger indels, a length of at least 20 bp of complementarity is better.^1^^,^^3^ These basic guidelines recommend testing at least nine pegRNAs for each locus, with three different PBS lengths and three different RTT lengths.^4^

The instability of the pegRNA can be prohibitive in successful gene editing. RNA degradation of the 3′ end of the pegRNA impairs editing efficiency, and many approaches have been tested to stabilize the 3′ end of the pegRNA. To mitigate the 3′ mRNA degradation, it is possible to add a pseudoknot to the 3′ end of the mRNA, which is a small RNA sequence with a defined tertiary structure. Two structures have been trialled so far: the Tevopreq_1_ motif and mpknot motif, which both improve the editing efficiency relative to an unmodified pegRNA.^43^ An 8 bp linker is essential to ensure that the tertiary structure of the pseudoknot does not interfere with the rest of the pegRNA structure. The web app pegLIT offers *in silico* design of a non-interfering nucleotide linker between a user-submitted pegRNA and the 3′ Tevopreq_1_ and mpknot pseudoknot motif. Currently, as a baseline, it is recommended to start with the Tevopreq1 motif, and test mpknot after optimization of the PBS, RTT, and spacer is satisfactory.

An alternative approach to stabilize the RNA is to use circularized pegRNAs. This was previously demonstrated to achieve up to ∼41% editing efficiency in HEK293 cells using a split nickase Cas12a PE2.^8^ In the literature, epegRNAs typically outperform pegRNAs, although the fold improvement is variable by gene locus and desired edit and can also vary by cell type.^1^^,^^3^^,^^6^^,^^44^ One study demonstrated that epegRNAs on average provided a 1.5-fold improvement in HEK293 cells with the PEmax system, and a 4.1-fold improvement in A549 cells, relative to pegRNA.^44^ This supports previous advice that epegRNAs should be favored over pegRNAs when designing PE experiments.^4^

One consideration when designing the RTT and PBS is the inherent complementarity of the spacer sequence and the primer binding sequence of the pegRNA, which may cause RNA-RNA interactions and inhibit the binding of the pegRNA-PE complex to target DNA sequences. Contradicting previous finding that a PBS of approximately 13 bp was optimal, Ponnienselvan et al*.* found that when using synthetic pegRNAs, a shorter PBS was favorable as it avoided auto-inhibition caused by the spacer and PBS binding, especially when optimized for a melting temperature of 37°C.^9^ Additionally, they demonstrated that a cold shock after PE delivery improved editing efficiency in primary T cells and zebrafish embryos. While further work testing the applicability of these findings to other models would be beneficial, this is a useful and important consideration when designing pegRNAs.

Another modification that can be made to the pegRNA structure is to change the scaffold by including a C-G at the base of the small hairpin, which improves the stability of the pegRNA, or by introducing additional same-sense mutations into the RTT at positions 1, 2, 3, 5, and 6.^45^ This can further improve the efficiency of the pegRNA.

The length of epegRNAs can impair their successful and efficient synthesis. A recent paper suggested synthesizing the epegRNA in two or three parts and then using a DNA splint and T4 RNA ligase to fuse the separate parts into an L-epegRNA. The 5′ and 3′ ends of the epegRNA were stabilized with three bases of 2′-*O*-methyl modifications and phosphorothioate linkages, increasing high-quality epegRNA synthesis and in turn increasing editing efficiency with PE mRNA and PE ribonucleoprotein (RNP).^46^

To design pegRNAs and epegRNAs for any PE approach, we would recommend using one of the developed tools available online. Multiple algorithmic tools exist such as pegFinder,^47^ PrimeDesign,^48^ PE-Designer,^49^ and well as machine learning algorithms such as DeepPE,^50^ PRIDICT,^51^ and most recently OPED.^52^ All these tools facilitate and improve pegRNA design; however, none offer a guarantee of editing efficiency, and the difference between expected editing efficiency and measured editing efficiency can be variable. It is not uncommon to try between 9 and 32 pegRNAs before finding a few with measurable editing efficiency.^10^ This is why it is paramount to design multiple epegRNAs and optimize within your model of choice.

### PE systems

Efficiency can be increased through the addition of different components and systems. This might be through the addition of an ngRNA (PE3),^1^ which introduces a break on the strand not targeted by the e/pegRNA to encourage the repair machinery to use the edited strand as the repair template. This increases editing efficiency at the cost of higher insertion deletions than without the ngRNA. Alternatively, mismatch repair inhibition can be used to enhance editing efficiency. It has been proposed that MMR either inhibits the 3′ flap re-annealing to the non-edited strand, or reverts the resulting flap-genome heteroduplex, and preferentially drives Exo-1-dependent or independent excision of the edited strand due to the nCas9 nick.^3^ This effect has been shown to be ameliorated via the addition of a dominant negative form of MLH1 (MLH1dn), yielding the PE4 strategy.^3^ Inclusion of both MLH1dn and an ngRNA yields the PE5 system, further enhancing editing efficiency.^3^

### Dual PE systems

PE facilitates not only small indels and base conversions but also large fragment modifications. One approach to support large fragment modifications is via dual PE systems, which use complementary pegRNAs to recruit two prime editors to encode larger fragment edits. Multiple dual pegRNA strategies were developed in a short period of time, each highlighting the advantage of a dual PE strategy over a single PE strategy.

The original dual PE system is twinPE, where dual pegRNAs with overlapping RTT sequences encode edits on opposite strands of the DNA to encode large indels.^11^ This eradicates reliance on endogenous repair mechanisms as it encodes complementary 3′ DNA flaps that anneal directly and avoid strand invasion. By removing the need for cellular repair factors, twinPE achieves higher efficiency and precision and lower indel formation. Large-sequence modifications including the insertion of recombinase recognition sites like attP was achieved using this strategy with efficiencies reaching 58%.^11^ TwinPE can also be used for single-base-pair substitutions, for example, in correcting the pathogenic variant in Shwachman-Diamond syndrome patient cells, where twinPE was used to achieve 8.7% editing efficiency, while a single-pegRNA strategy only achieved up to 2.7%.^12^

Bi-PE employs a similar approach, inspired by indications that positioning the ngRNA in close proximity to the homology arm (HA) improves large fragment deletions in PE3 systems, engineering the ngRNA into a second pegRNA. In contrast to twin-PE, Bi-PE ensures more overlap between the pegRNAs to cover the nicking site and the 5′ flanking regions.^13^

To test the efficiency of Bi-PE in large genomic deletions, nine targeted deletions were conducted across four loci (*β-Actin*, *VEGFA*, *AAVS1*, and *DMD*) in HEK293 cells. At the *β-Actin* locus, Bi-PE achieved an average deletion efficiency of ∼11.3%, which was 1.53 times higher than PE3. For a 654-bp deletion, Bi-PE was on average 10 times more efficient than PE3. The 861-bp deletion efficiency reached ∼31.5%, a 4-fold increase on average over PE3. Multiplex base conversions also showed improvement, but single-base editing remained comparable to PE3. The overall undesired indels were shown to be considerably lower in Bi-PE system compared to conventional PE.^13^ Similarly to Bi-PE, PRIME-Del takes advantage of paired pegRNAs and generates precise deletions of up to 10 kb with 1%–30% efficiency by using paired pegRNAs. In addition to deletions, it can insert small sequences such as protein-encoding fragments known as “epitope tags” at the deletion site, which allows tracking proteins intracellularly. By avoiding DSBs, PRIME-Del reduces unwanted editing outcomes, which makes it a reliable tool for precise genome modifications.^14^

HOPE employs a similar strategy, but is optimized for smaller sequences, by selecting PAM sites up to 50 bp apart and improves large fragment modifications by utilizing paired pegRNAs, with PAMs facing toward each other facilitating edits on both DNA strands in tandem. This strategy considerably enhanced editing efficiency and achieved 1.8- to 3.5-fold improvements over PE2 with indels reduced 11-fold compared to PE3.^15^

PEDAR uses a dual pegRNA strategy but instead adopts a fully active and DSB-inducing Cas9 nuclease rather than nCas9 conjugated to an RT. This approach was shown to generate precise large-fragment deletions and insertions with 17.7% efficiency at the *HEK3* locus, though at the cost of potential off-target effects due to DSB formation.^16^

Like PEDAR, PE-nuclease-mediated translocation and inversion (PETI) was developed to induce chromosomal translocations and inversions. By using paired PE2 nucleases along with paired pegRNA, PETI induces two DSBs, and then encodes 3′ flaps on each DSB, which are complementary to the other target site. This has been demonstrated to result in up to 78% cancer-specific translocation efficiency, surpassing SpCas9.^17^ This approach would allow more complex genome engineering to model disease phenotypes.

GRAND editing uses a similar dual pegRNA approach, although has less overlap in the complementary 3′ region, and is intended for large insertions. Deep sequencing revealed that accurate editing efficiency was 42.7% for a 101-bp insertion with a net size gain of +48 bp due to the simultaneous insertion of a synthesized 101-bp strand and 53-bp deletion of the original DNA strand. The efficiency remained high at 43.7% for 150-bp insertions but decreased to 7.6% for 200-bp insertions, which suggests a size-dependent decline in accuracy.^18^

The extended prime editor system (EXPERT) uses a dual prime editor approach instead of a dual pegRNA approach, which includes a critical modification to pegRNA structure that facilitates a novel editing approach.^19^ Two PE fusion proteins are used in each edit. One PE protein binds the pegRNA as in canonical PE, with the nCas9 binding the spacer sequence and the RT binding the RT template. The second protein fusion’s nCas9 binds a ngRNA (upstream sg-RNA [ups-sgRNA]) targeting upstream of the pegRNA’s spacer, while its RT binds an extension of the pegRNA. The extension of the pegRNA contains a secondary PBS, which binds the 3′ flap generated by the ups-sgRNA cut. Cutting of both nCas9s generates two nicks on the same DNA strand. The gap between these nicks can then be filled in via activity of the two RTs, facilitated by the two PBS sites flanking the RTT. Therefore, EXPERT can edit upstream of the canonical pegRNA cut, a feat unattainable with other PE strategies. EXPERT was compared with PE2 to generate a 40-bp replacement at the *HEK4* site, and outperformed PE2 by 122-fold, achieving 6.1% editing efficiency and a comparable rate of unwanted indels.

Gene duplication is a major driver of increasing genetic diversity and evolution, so recreating gene duplication events is desirable in genome engineering. Two PAM sites are selected to encode edits as in other dual PE strategies, but whereas in twin PE, the RTTs are facing toward each other, and in amplification editing [AE] the edits are distal to each other but are complementary. These edits hybridize, forming a loop that contains the region to be duplicated. During DNA synthesis, this loop is then synthesized twice, with a small inserted sequence between. AE can duplicate regions of up to 100 Mb, which is valuable for disease and evolution research, and has been demonstrated to insert duplications of up to 70% efficiency in HEK293 cells, although efficiency decreased with duplication size.^53^

To summarize the similarities and differences between the dual PE systems, all except EXPERT employ a dual pegRNA strategy, whereas EXPERT employs an ngRNA and a modified pegRNA with dual PBS sites. PEDAR and PETI utilize nuclease prime editors to induce DSBs while the edits on the RTs are encoded. Bi-PE and Prime-DEL are optimized for large deletions, and Bi-PE has larger overlapping regions in the pegRNAs to include nicking site and 5′ flanking regions. HOPE uses PAM sites that are < 50 bp apart to encode slight edits, and twinPE can encode larger indels. AE is appropriate for causing gene duplications at the chromosomal scale. Depending on the exact edit desired, different strategies may be selected, but no widescale study has compared all dual PE strategies to indicate the optimal approach in each use case.

### PE-integrase combination technologies

Another recent advancement in the DNA editing field has been the use of PE to introduce recombination motifs for virus-derived serine integrases, allowing for genomic integration of multi-kb DNA donors.^54^^,^^55^ PASTE and PASSIGE (Figure 1) function on near-identical principles: PE (twinPE in PASSIGE) is used to install a ∼50-bp attP or attB motif into the genome at the target locus, and a serine integrase protein is introduced alongside a DNA donor carrying the corresponding attB or attP motif (respectively). The serine integrase then recombines the donor molecule into the genome. This approach allows installation of entire genes into the genome using PE’s Cas9-guided precision. Whole-gene insertion represents a variant-agnostic treatment strategy for monogenic disorders with many causative mutations. In its initial reporting, PASTE was used to integrate *HBB* cDNA at the *ACTB* locus with 16% efficiency,^54^ while PASSIGE was used to integrate *FANCA* cDNA into HEK293 cells with 46% efficiency.^55^ There are three principal differences between the two technologies. First, in PASTE, the integrase and PE architecture can be delivered as a split or fused protein; in PASSIGE, the two architectures are delivered as separate molecules. In PASSIGE, the placement of attP and attB in the donor or genome is variable based on target genomic locus; in PASTE’s initial reporting, only attP as the genomic targeting motif and attB in the donor were explored. Finally, the integrase used in PASSIGE has been engineered and evolved to achieve up to 16-fold higher integration efficiency than the integrase used in PASTE.^55^

## Delivery methods for PRIME editing machinery

As described in the previous section, delivery of PE machinery is mainly achieved in the form of plasmid DNA (pDNA) or mRNA. There are several major methods for delivery. The most common delivery methods are electroporation, viral delivery, lipid nanoparticles (LNPs), and engineered virus-like particles (eVLPs).

### Electroporation and nucleofection

Electroporation uses a brief electric pulse to generate temporary pores in a cellular membrane by inducing a voltage differential on either side of the cell membrane.^56^ The exterior of the cell is negatively charged while a positive charge accumulates on the interior of the temporary pore. This voltage differential draws negatively charged pDNA or mRNA into the cell. Once the electrical pulse is ended, the pores close, restoring membrane integrity, and transcription and translation can occur.^57^ Nucleofection is an extension of this principle, by using electrical pulses to create pores within the nucleus as well as the cytosol, therefore enhancing transfection efficiency in hard-to-transfect cells such as primary cells. Electroporation is highly efficient; however, it has a higher cost to entry than other approaches, consequences for cell viability, and has reduced relevance for *in vivo* delivery.

### Viral delivery

Using viruses to deliver gene editing machinery is a popular approach. The most common viral vectors are adeno-associated virus (AAV) approaches, which require splitting the PE protein across two AAVs. When a split PE2 protein in AAV was delivered to HEK293 cells targeting the *HEK3* locus, 27% editing efficiency was achieved.^20^ After optimization in HEK293 cells at the *HEK3* locus, this split PE approach was delivered into mice retinas to correct retinal degradation, significantly increasing the mice’s vision.

In animal models, different split locations for AAV approaches have been tested, such as the V1em and V3em variants, which have been successful for correcting phenylketonuria-causing mutations *in vivo*,^21^ achieving 41% correction in the mouse liver.^22^ Both approaches have resulted in high-efficiency editing in mice and show potential. However, the dual AAV approach comes with the risk of undesired expression products,^58^ which needs to be tested extensively prior to clinical use.

Typically, PE proteins must be split in dual AAV approaches; however, with the use of high-capacity adenoviral vector particles, the whole prime editor can be encapsulated. This was elegantly demonstrated to result in high editing efficiency in Duchenne muscular dystrophy myoblasts, achieving up to 70% correction of the pathogenic variants without selection.^23^

Lentiviral delivery has higher immunogenicity than AAV vectors, but offers a higher payload capacity, and is desirable for *ex vivo* gene therapies. For example, by using a dual lentivirus strategy to deliver PEmax into stem Leydig cells CD51+ W495X-SLCs, it was possible to correct a *Lhcgr* W495X variant by 23.43%. After transplanting the cells back into the testes of Lhcgr-deficient mice, hereditary primary hypogonadism could be completely rescued.^24^

Viral delivery has some disadvantages, such as higher immunogenicity than other methods, risk of insertional mutagenesis, and uncontrolled release, which is why non-viral delivery approaches such as LNPs and eVLPs are becoming increasingly desirable.

### Lipid nanoparticles

LNPs are attractive options for both *in vivo* and *ex vivo* applications due to their low toxicity, high efficiency, and modularity. Lipofectamine is a common LNP used for gene editing, but custom LNP formulations offer attractive benefits. Inclusion of differing lipid species, as well as supplementation with “helper” lipids, have been shown to offer enhanced efficiency. For example, cationic or ionizable lipids interact favorably with anionic nucleic acid cargo and cell membrane components^59^^,^^60^; neutral phospholipids aid in endosomal escape^61^^,^^62^^,^^63^; cholesterol lends structural stability^64^; polyethylene glycol (PEG) increases circulation time and allows coating of LNPs by apolipoprotein E (ApoE), which aids in cellular uptake.^65^ Inclusion or exclusion of different lipid species, particularly PEG, has also been shown to alter whether LNPs are uptaken by cells via micropinocytosis versus clathrin- and caveolae-mediated endocytosis.^66^ LNPs have been used in PE studies to deliver PE machinery as both nucleic acids and RNPs.

In 2020, a study utilized the ionizable lipid DLin-MC3-DMA, the phospholipid DSPC, DMG-PEG-200, and B-sitosterol (substituted for cholesterol), yielding engineered LNPs (eLNPs) that achieved up to 54% editing efficiency in a reporter cell line when encapsulating PE3 mRNA, a 7-fold improvement over LNPs formulated with cholesterol.^65^ An MC3-based ionizable lipid was also used *in vivo* in 2023 to encapsulate chemically modified pegRNAs and PE2 mRNA for retroorbital injection into mice, yielding up to 5% editing of the *Pcsk9* gene.^67^ Weekly repetition increased editing rates to 8%. However, it is important to note that the liver naturally accumulates intravenously injected materials, and this formulation likely yielded significantly lower editing rates in other tissues (analysis of non-liver tissues was not reported). Editing was significantly enhanced by use of a different ionizable and helper lipid species (306-O12B instead of MC3 and DOPC instead of DSPC).^67^

A late 2024 study from the Liu group reported that LNPs consisting of the ionizable lipid SM-102 and 2.5% DMG-PEG-2000 successfully delivered PE2-RNPs for correction of a nonsense mutation in the disease-relevant gene *RPE65.*^68^ Correction was achieved in an *rd12* reporter cell line (>20% correction, significantly outcompeting non-PEGylated SM102 LNPs), and via subretinal injection in an *rd12* mouse model of Leber congenital amaurosis (0.12% correction), with no detected off-target effects.^68^

### Engineered virus-like particles

Briefly, eVLPs function via assembly of packaging protein components produced by cells but lack a packaged genome, allowing for packaging and cellular transduction of varied cargo including RNPs.^35^ Typically, cargo of interest is included as a fusion with a Gag protein, which interacts with VSV-G to form an intact particle. Between Gag and RNP, several elements are included: a nuclear export signal (NES), which allows released Gag-RNP fusion protein molecules to localize to the cytoplasm; an engineered protease cleavage site, which allows RNP to be decoupled from Gag; and an NLS, which allows decoupled RNP to re-localize into the nucleus for DNA editing. A 2024 report by the Liu group detailed the development of novel eVLPs for delivery of PE-RNPs.^35^ Several enhancements to eVLP architecture were reported. A protease cleavage site was theorized to be unfavorably close to the cargo PE-RNP’s NLS and was therefore removed; the NLS was moved to a site at which Gag was more efficiently decoupled from PE-RNP; a linker was included on either side of the protease cleavage site in order to enhance decoupling of Gag and PE-RNP. Additionally, epegRNA packaging was enhanced via the inclusion of an MS2 loop in the epegRNA scaffold, paired with an MS2 coat protein included in eVLP architecture. These improvements yielded v3 PE-eVLPs. Further alterations, including using a P3-P4 coiled coil peptide pair to couple PE-RNP to Gag without production of a protein fusion and replacing the P3-P4 peptide with a COM-Com protein-RNA aptamer pair, were found to enhance or not enhance editing efficiency in a locus-specific manner.^35^

Upon completion of an editing experiment in any model, the next step is to assess on-target editing efficiency. Several reporter technologies have been developed to detect successful PE events either *in vitro* or *in vivo.*

## Delivery and models

A few main methods of delivering PE are currently in use. PE plasmid or mRNA are delivered via lipofection or electroporation, or the PE and pegRNA are packaged into viral vectors. Some research has used PE RNP complexes,^9^ but as PE protein is not yet available commercially and is challenging to synthesize in house,^69^ this approach is less common. Delivery methods are highly model dependent, and what works for one model may not be suitable for another.

### Cell lines

In this section, we will consider the common approaches used to prime edit cell lines. It is common in PE to use cell lines to optimize e/pegRNA design for a target locus.^4^ HEK293 and variant cell lines are typically considered easy-to-transfect workhorse cell lines and so are ideal for optimizing e/pegRNA design before bringing PE systems into disease models.^70^ As HEK293 cells are easy to transfect with large plasmids and amenable to transfection by the commercially available lipid-based transfection reagent Lipofectamine and other lipofection approaches, there is a low barrier to entry using HEK293 cells as models. Additionally, starting with plasmids allows for quick modification and alteration to the e/pegRNAs and PE plasmids themselves.

Selection can be used to enhance editing efficiency where it is unlikely to do harm. Oliveros et al. used the PE3 plasmid system, with pegRNA design optimized with DeepPE, delivered with Lipofectamine 3000, to induce blood-pressure-related variants identified by GWAS, across 32 pegRNAs across various loci.^10^ By using a puromycin-resistant cassette in their plasmids, they were able to select from successful transfection with three of their pegRNAs and then used single-cell expansion to generate clones with ∼30%, ∼60%, and ∼100% variant alleles. By having precisely selected ratios of gene variants, it was possible to further elucidate the roles of these variants in blood pressure and hypertension. This demonstrates the power of selection and single-clone expansion to generate cell lines with the desired mutation, although this may result in bottleneck effects with spontaneous mutations.

Cell line models are also well suited for optimizing new delivery approaches and novel protein structures to improve efficiency. Lentivirus-derived nanoparticles were engineered to encapsulate various gene editing tools, including PEmax and PEmaxΔRH (a PE protein with no RNAse activity). Proteins were delivered via RNP pre-complexed with epegRNAs. Targeting the *HEK3* locus with PEmax and PEmaxΔRH, researchers were able to insert a CTT sequence with 6% and 5% efficiency, respectively, in HEK293 cells,^71^ confirming the potential of using viral-derived particles for delivery without the risks associated by viral loads.

It is important to note that even within cell lines, there is considerable variability in editing efficiency dependent on the locus, and in some studies, it has not been possible to achieve PE even in cell lines. Once PE is successful in your chosen cell line, it is time to bring it to your relevant model of choice.

### Primary cells

Primary cells allow for PE techniques to be tested in relevant models, often derived directly from patients. However, they are more variable from passage to passage, more sensitive to cell culture perturbation, and typically more difficult to transfect.^72^ In the studies looked at for this review, electroporation was more common than lipofection for delivery. There are more options for electroporation optimization than lipofection methods, with specific kits often available designed for specific cell types. PE mRNA was also used frequently, as it is typically easier to transfect than large plasmids, and often results in higher editing efficiency.^73^ However, mRNA is expensive to synthesize, and due to the size of the PE mRNA, it can be difficult to synthesize without abortive variants.

Increased rates of editing in primary cells can be achieved by multiple doses of PE machinery. An example of this is demonstrated by Godbout and colleagues, targeting motor disabilities caused by mutations in the *RYR1* gene, specifically the T4709M variant. After optimization of the pegRNAs and ngRNAs to target the T4709M in HEK293 cells, electroporation was used to deliver PE3 plasmid and mRNA to primary myoblasts and primary fibroblasts bearing the variant. Plasmid transfection resulted in 10% editing efficiency, whereas mRNA delivery resulted in over 30%. By multiple rounds of PE electroporation, the final editing efficiency was up to 59%.^25^

### Stem cells

Stem cells pose unique challenges as sensitivity to cytotoxic compounds can trigger cell death or differentiation, resulting in a loss of stemness.^74^^,^^75^ However, they are desirable targets for gene editing to create pools of cells with long-term viability. They are also highly desirable targets for potential gene therapies, as they maintain cellular populations in organs and tissues long-term, reducing the need for repeat treatments. Similarly to primary cells, electroporation is the most used approach for delivering PEs to stem cells, and mRNA and plasmid are both commonly used (Table 1).^26^^,^^27^^,^^76^

Most PE work so far has occurred in induced pluripotent stem cells (iPSCs).^77^ By differentiating isogenic populations of iPSCs into multiple cell types, it is possible to identify the impact of a variant in a wide range of biological contexts.^78^ In a study using PE to create a disease model, PEmax mRNA was electroporated into wild-type iPSC lines to introduce six variants associated with type 2 diabetes. After extensive pegRNA optimization and selecting for successful transfection, up to 73% editing efficiency was achieved.^76^ This paper demonstrates an iPSC PE pipeline that is achievable in a 4- to 5-week period.

As discussed previously, selection and single-cell expansion can be crucial to optimizing PE in iPSCs. iPSCs are particularly suited for this due to their capacity for forming colonies from single cells. By utilizing this strength, it is possible to generate iPSCs lines with the desired edit. Binder et al.*.* induced beta actin loss-of-function mutations in wild-type iPSCs, using PEmax-GFP-tagged plasmid, delivered with Lipofectamine stem. By selecting for GFP expression and then subcloning, isogenic lines were developed, which can be used to study the impact of beta actin haploinsufficiency.^26^ Instead of GFP selection, Sib selection and puromycin selection offer alternative selection pressures without the need for flow cytometry. PE3 was electroporated into cystic-fibrosis-patient-derived iPSCs with a homozygous *CFTR* W1282X variant. Initial low (0.36%) editing efficiency was increased to 3.4% after selection. This allowed corrected clones to be isolated, and then differentiated into organoids, which demonstrated a rescue of the CF phenotype.^27^ Selection pressure can greatly improve efficiency in editing iPSCs, when introducing additional vectors is appropriate, which it may not be for therapeutic purposes.

Outside of iPSCs, PE has also been used in hematopoietic stem and progenitor cells (HSPCs). After optimizing PE3 pegRNAs and epegRNA editing in K-562 cells to knock out β_2_-microglobulin, PE3 mRNA was electroporated into mobilized peripheral blood HSPCs, resulting in up to 40% editing, with 4.5% unintended indel rate. Additionally, when compared to Cas9 editing of the same locus, prime-edited HSPCs showed lower impact of clonogenicity but higher activation of pro-apoptotic pathways. Edited HSPCs were successfully transplanted into mice without loss of editing. This demonstrates the value of PE for HSPCs and that high efficiency can be achieved but that there may be long-term cellular consequences.^28^

Altogether, high editing can be achieved with PE in stem cells, but more work needs to be done to demonstrate applicability in a wider range of stem cell populations. Here as well, electroporation and mRNA appear the preferable way forward, although plasmids have been used successfully.

### Animal models

Less PE so far has been done in mammalian animal models, although more animal-based reports have been published in the last couple years. Animal models are essential for bringing PE to potential clinical application. Viral and viral-derived particle deliveries *in vivo* have been the preferential approach thus far in the literature,^29^ due to the high efficiency of transduction. High efficiency of delivery is especially crucial in animal models as it is rarely possible to apply a selection pressure during *in vivo* treatment.

Instead of relying on *in vivo* approaches, *ex vivo* gene editing followed by grafting allows for greater control over editing efficiency. It allows cells and tissues to be checked for editing, deleterious off-targets, and changes in transcriptome and proteome expression prior to engraftment back into animals. However, the pipeline for *ex vivo* editing is far more complex. First, cells must be extracted from the relevant tissue, then edited, checked, and then surgically re-engrafted into the animals. Some target tissues are easier to re-engraft than others. As previously described, dual lentiviral approach has been used to prime edit CD51+ W495X-SLCs in an *ex vivo* capacity, which could then be transplanted back into Lhcgr-deficient mice.^24^ Conversely, less accessible internal tissues may be more difficult to engraft without prohibitively invasive procedures, especially if an individual’s overall health is already damaged by disease.

There are advantages and disadvantages to *in vivo* vs. *ex vivo* delivery as discussed, so both must be weighed up extensively depending on the needs of the specific treatment. There is very little literature testing PE in larger mammals, which will be the next step in bringing these therapies to the clinic.

## Reporters of PE efficiency

Quantification of editing efficiency is the standard readout of success for any CRISPR-based experiment. Next-generation sequencing (NGS) is regarded as the “gold-standard” method of measuring changes at the nucleotide level.^7^ However, various techniques have arisen in recent years that use markers and reporters to assay editing events. These reporters rely on mutated, nonfunctional reporters of fluorescence or luminescence; successful editing restores signal, allowing for detection of cells in which PE has been successfully executed.

Reporters rely on editing of the reporter construct itself, rather than the on-target genomic sequence. Therefore, they fall into two categories. The first consists of approaches that require targeting a sequence unique to the reporter construct itself, which are best suited to optimizing more general aspects of PE (i.e., transfection efficiency, general principles of pegRNA/gRNA design, etc.). The second category consists of approaches that allow flexibility in gRNA design, meaning that researchers can test bespoke strategies (i.e., generation or correction of specific disease-relevant alleles).

### Methods for general PE experimental design

The technology prime-induced nucleotide engineering using a transient reporter for editing enrichment (PINE-TREE) utilizes a construct that expresses blue fluorescent protein (BFP) when un-edited but expresses green fluorescent protein (GFP) upon installation of a C>T edit.^79^ In addition to reporting successful editing, this system can also report indels via loss of any fluorescence. In its initial report, a HEK293 cell line was generated to endogenously express the TREE reporter construct.^30^ Subsequent transfection with PE reagents and use of PINE-TREE to sort for cells in which editing had occurred, rather than merely sorting for GFP signal after co-transfection with a GFP-expressing plasmid, yielded greater enrichment of successfully edited cells.

For optimization of PE efficiency *in vivo*, a late 2023 study developed a traffic light reporter (TLR) mouse model, deemed TLR-multi-Cas variant 1 (TLR-MCV1).^80^ In the TLR-MCV1 reporter, a CAG promoter drives a cassette containing mutant GFP and mCherry constructs separated by a T2A motif. The GFP construct is mutated in a manner fixable only by precise DNA repair (such as by PE or HDR); the mCherry construct is frameshifted in a manner fixable by precise or imprecise (indel) DNA repair. Using this reporter, PE was assessed *in vivo* using a mouse model with the TLR-MCV1 construct incorporated (via CRISPR/Cas9 + HDR) at the *Rosa26* genomic safe harbor locus. Editor and pegRNA plasmids were injected into the tail vein, and 4 bp insertions were detected in ∼2% of assayed liver cells via amplicon NGS. Using immunohistochemistry staining for mCherry expression, mCherry+ cells were detectable in liver tissue.

### Methods for custom pegRNA design validation

#### Prime editor activity reporters

The prime editor activity reporters (PEARs) developed by the Welker group offer fluorescent output via treatment with custom pegRNAs.^31^^,^^81^ PEAR relies on a GFP cDNA split by an intron; the intron contains a splice donor site mutated to be inactivated. Splicing is restorable by PE editing; therefore, successful editing restores fluorescence.^31^ Crucially, the intronic sequence acting as the spacer can be custom designed to match any genomic target locus, as PE’s editing window is not restricted to falling within the spacer sequence.

When a PEAR-expressing plasmid is transfected into cells alongside PE machinery, restoration of fluorescence is taken as a signal that editing has successfully occurred. Used thus, this reporter functions episomally. A direct comparison between episomal and integrated editing efficiency is difficult due to the potential confounding factors of transfection efficiency and plasmid copy number. Indeed, copy number was suggested as a potential explanation for the discrepancies between the editing efficiencies observed with the PEAR system.^31^

#### Bioluminescence resonance energy transfer assay

In 2023, Wimmer et al. reported use of a bioluminescence resonance energy transfer (BRET)-based assay to detect editing events in a disease-relevant gene in HEK293 cells.^32^ A section of the gene, including the frameshift-causing single-base insertion, was cloned in-frame in a BRET reporter system. The construct was inserted in the reporter construct between RLuc8 and GFP2 coding sequences, producing a shift in the GFP2 reading frame and eliminating green fluorescence. The PE2 system was applied with 27 distinct pegRNAs. Plasmids encoding the BRET construct, the PE editors, and custom RNAs were transfected into HEK293 cells, and cell lysate was characterized via the BRET assay. Successful editing of the target gene construct rescued expression of the downstream GFP2 reporter, thereby restoring fluorescence. The reporter was found to overestimate editing efficiencies when compared with tracking of indels by decomposition (TIDE) analysis; however, trends in compared pegRNAs were consistent across both methods. Overall, the BRET method allows for more flexibility in experimenter-designed pegRNAs for specific disease alleles and appears to accurately readout trends in editing efficiency if not precise quantification.^33^

Despite this promising correlation, it is important to note that any assay functioning episomally does not reflect editing efficiency in the native chromatin environment. These approaches are therefore best suited to narrowing libraries of pegRNAs/ngRNAs to a smaller number of candidates to validate in the genome. Once on-target editing efficiency has been established for the locus of interest, the next step is evaluating the safety profile of the editing strategy. This involves assessing off-target editing events (usually specific to individual pegRNA sequences) and characterizing the effect of PE events on a specific cell or organism’s overall health.

## Novel methods to assess off-target effects

In addition to optimizing on-target efficiency, it is equally critical to accurately assess off-target effects (OTEs). Traditional methods like GUIDE-seq and nDigenome-seq provide indirect measures of off-target activity. GUIDE-seq measures Cas9-induced DSBs by incorporating double-stranded oligodeoxynucleotides (dsODNs) at the break sites.^82^ The regions where these tags are integrated are then mapped using targeted amplicon sequencing. In contrast, nDigenome-seq is designed to detect single-strand breaks induced by nCas9, following an in-vitro digestion of genomic DNA with nCas9.^83^ This method relies on high-throughput sequencing to map the positions of DNA nicks across the genome, allowing for the identification of both on-target and off-target activity of nCas9.

However, a novel approach called TAPE-seq (Tagmentation of prime editing sequencing) has been introduced, which directly measures off-target effects of PEs in live cells. In TAPE-seq assay, PE- and pegRNA-encoding vectors are integrated into the genome using PiggyBac transposase system, incorporating a 35-nt tag between the PBS and RT template in the pegRNA. This is followed by tag-specific amplification of regions with PE outcomes to map and quantify on- and off-target effects specific to PE offering several advantages over existing methods, including fewer missed off-target sites, higher predictive accuracy, and fewer false positives. This method’s unbiased, cell-based approach, which accommodates the reverse transcription mechanism of PE, provides a more accurate reflection of PE’s off-target effects, making it a valuable tool for optimizing editing protocols and ensuring safety.^84^

## Safety concerns and undesired effects

PE has shown great potential for precise DNA modifications. However, understanding its genotoxic effects, efficiency variations, and off-target risks is crucial for its safe and effective application. Recent research on PE in HSPCs has provided valuable insights into these challenges and opportunities for optimization.

### Genotoxic effects and PE efficiency

One study focused on the PE3 system, a version of PE that utilizes a ngRNA, aimed to knock out β2 microglobulin (β2M) encoded by the *B2M* gene in HSPCs derived from mobilized peripheral blood (mPB) cells. This approach was chosen because β2M is a component of the major histocompatibility complex (MHC) class I complex, which is ubiquitously expressed on the surface of all cell types. Editing efficiency was assessed through flow cytometry by quantifying loss of β2M expression. Deep sequencing of the target site showed a 40% on-target editing efficiency, which is promising for therapeutic applications. However, there was also a 4.5% undesired editing outcome, characterized by the integration of the first bases of the pegRNA scaffold into the target site or the occurrence of small indels at the nicking sites. These findings highlight that even with targeted precision, PE can introduce unintended genetic changes, necessitating careful evaluation of both on-target and off-target effects to minimize potential genotoxicity. Moreover, transcriptomic analysis conducted 24 h post-PE3 editing showed significant upregulation of genes associated with interferon signaling (such as *ISG15* and *IFI6*), p53 activation (including *MDM2* and *CDKN1A*), and the unfolded protein response (like *ATF3* and *HSPA5*). This suggests that PE induces a cellular stress response, which could impact editing efficiency and cell viability.^28^

### Influence of chromatin context and epigenetic modulation

The influence of factors such as endogenous DNA repair pathways, edit types, and RTT and PBS lengths on PE efficiency has been more extensively studied compared to the impact of cis chromatin at target loci. To investigate this, a PiggyBac-transposon-based PE reporter library containing a PE target was synthesized and randomly integrated throughout the genome of K562 cells that had constitutive expression of PE2. After transfection with reporter-targeting pegRNA, reporter sequences positioned differently across the genome exhibited a wide range of editing outcomes, indicating that *cis*-chromatin features may influence PE editing efficiency. Regions with active transcription, marked by histone modifications like H3K79me2, showed higher editing efficiencies. Researchers demonstrated that using CRISPRa—which unlike traditional CRISPR/Cas9 system, takes advantage of transcriptional activators fused to catalytically inactive Cas9 enzyme (dCas9)—to activate transcription at target loci before editing significantly increased PE efficiency, while silencing target genes using an epigenetic silencing system CRISPRoff reduced editing outcomes by 39%–47%. These findings indicate that optimizing PE could involve targeting regions with favorable chromatin states or using epigenetic modulators to enhance editing efficiency in less accessible chromatin environments.^34^

#### Impact of RTT design on prime editing specificity and efficiency

In a different study, researchers assessed PE2 off-target effects using a tagmentation system, examining how varying the lengths of the Tag sequence and homology arm within the RTT sequence influenced these effects.^85^ Given that previous findings showed reverse transcription of the RTT could still proceed despite modest mismatches between the PBS and the 3′ end of the nicked non-target sequence, it was crucial to investigate how the lengths of individual RTT components affected outcomes. Using epegRNA targeting the *HEK4* locus in HEK293 cells, the study observed mixed effects when increasing the Tag sequence length, while increasing the homology arm length from 7 to 20 nucleotides consistently reduced off-target activity and improved on-target editing efficiency.

#### Impact of DNA topology and supercoiling

Understanding the factors that influence off-target effects is vital for refining PE techniques. One significant factor is DNA topology, which refers to the study of the spatial arrangement and coiling of DNA in three-dimensional space, which influence its biological functions like replication and transcription. Studies have shown that negative DNA supercoiling promotes Cas9 off-target activity by increasing DNA accessibility and facilitating R-loop formation due to DNA duplex destabilization by supercoiling. This makes the DNA sequence more prone to unwinding and allowing Cas9 to bind and cleave at off-target sites. As PE also requires R-loop formation to operate, it is conceivable that DNA supercoiling could similarly impact PE specificity.^86^^,^^87^ Moreover, negative supercoiling prestresses DNA double-helix, which results in R-loops being formed more easily that in turn could influence how effectively PE engages its target sites.^88^ In line with this, TOPO-seq, which is a method to assess genome-wide Cas9 activity in different DNA topological contexts, has shown that underwound or negatively supercoiled DNA significantly increases the number of off-target sites cleaved by Cas9. Also, when base editors like ABE8e, built on the nickase Cas9, just like prime editors, were used in human cells, many of the nominated topology-sensitive off-target sites showed substantial editing in HSPCs. In some cases, off-target editing reached over 30% and persisted *in vivo* after 16 weeks. This suggests that off-target activities of nCas9-based editors such as PE are influenced by local DNA spatial contexts. Overall, these findings highlight the importance of assessing DNA structure and not just sequence, to predict and minimize off-target effects in prime editing applications.

#### Balancing precision and efficiency

**Figure 2:**
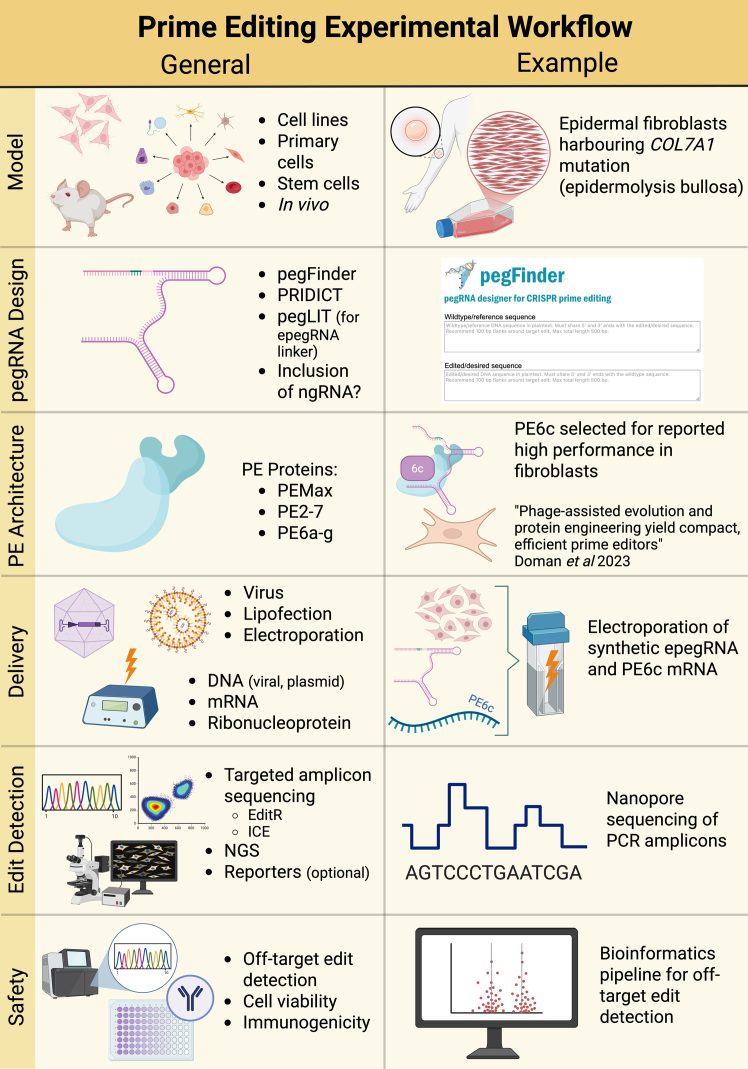
Illustration of a generic and specific prime editing experimental workflow Left and right differentiates the generic and specific prime editing experimental workflow. The following steps are illustrated: selection of a model, design of pegRNAs (and ngRNAs, if desired), delivery formats and methods, detection of successful editing, and assessment of safety. Examples of online tools for pegRNA design (pegFinder,^47^ PRIDICT,^51^ and pegLIT^43^) and edit detection (ICE^89^ and EditR^90^) are listed in the left-hand panels for the corresponding stages of workflow. NGS, next-generation sequencing; ICE, incidence of CRISPR dits.

Achieving a balance between precision and efficiency is a central challenge in the development of PE technologies. The interaction between PEs and chromatin context, along with factors like DNA topology, must be carefully managed to optimize outcomes. For example, a 10-fold upregulation of the *MT2A,* overexpression of which has been associated with apoptosis, was observed post-PE3 editing.^28^ This upregulation was shown to depend on both the active RT and the DNA nicking performed by the nCas9 component of the PE3 system. Further analysis revealed that *TP73* upregulation did not occur in cells transfected with catalytically inactive RT fused to nCas9, suggesting that the editing-induced proapoptotic stimulation required both components to be active.^28^ These insights guide structural changes to the RTT of PEs that maintain high editing efficiency while minimizing undesired effects. Combining these strategies with advanced predictive tools and cell-type-specific assays like TAPE-seq will be essential for developing reliable and safe PE protocols (Figure 2).

The findings from these studies emphasize the need for comprehensive undesired effect screening, particularly in genomic regions characterized by high transcriptional activity or negative supercoiling. Copy-number variation (CNV) analysis revealed that PE3 editing did not cause significant changes in the number of *B2M* copies compared to unedited cells, which is reassuring for maintaining genomic stability. However, strategies involving the development of PEs with enhanced specificity, coupled with epigenetic conditioning to create favorable chromatin environments, are necessary to improve the reliability and safety of PE for clinical use. Such advancements will pave the way for the broader adoption of PE in therapeutic settings, making precise and safe genetic modifications a reality.

Taken together, we recommend the careful consideration of which model will be most beneficial for the study of interest (Figure 2). The model will indicate which form of delivery is most optimal—while plasmid and lipofection will be sufficient for cell lines, for *in vivo* work, viral delivery is likely to be preferable, as discussed in our models’ sections. Extensive tools exist to design the optimal pegRNA, such as pegFinder, and synthesis of the epegRNA in multiple parts with ligation and appropriate chemical modifications will improve editing outcomes.^46^ Growing options for PE architecture and strategy must be fully considered, with different architectures offering advantages for different mutations and model types. Based on the decisions made thus far, delivery can be optimized, and editing detection can be achieved with targeted amplicon sequencing, or NGS, although reporters offer a rapid detection method for optimization. Finally, as with all gene editing, off target detection, viability, and immunogenicity must be considered.

## Discussion

Prime editing is an increasingly vital tool in both basic and translational research. The technology is advancing rapidly, with multiple research groups developing novel variations in protein architecture in the past year alone.^5^^,^^6^ The theoretical modularity of PE approaches (mismatch repair inhibition, secondary ngRNA, inclusion of RNA binding protein La, and use of evolved RT domains) has yet to be systematically tested to its full potential in different combinations. For example, the PE7 approach was developed using PEmax RT, while PE6 has developed multiple smaller RT domains for use in different cell types. Additionally, no reports have yet emerged of PASTE/PASSIGE being performed using the PE7 approach. At this stage, most publications have relied on PEmax; however, it is reasonable to anticipate that PE6 and PE7 (or some combination therein) will become increasingly popular in the near future. Researchers should consider all possible proteins when designing a new prime editing experiment (Figure 2).

Another major hurdle to PE optimization is the lack of commercially available PE proteins. The more recent PE architectures are commercially available solely as plasmids. *In vitro* transcription can be used to generate mRNA, but *in vitro* protein synthesis is more time- and resource-consuming. The aforementioned high pace of optimization and modularity of fusion protein architectures may reduce the likelihood of companies’ mass-producing pure protein in the near future. Therefore, mRNA delivery will likely continue to dominate the field.

Multiple design tools are available for algorithmic generation of epegRNAs. A head-to-head, experimentally validated comparison of these technologies has yet to be reported by an independent party. At the moment, a viable design strategy is to use multiple design tools and identify pegRNAs predicted by multiple platforms to have high efficiency and low off-target effects. Additionally, inclusion of silent mutations in the RT template to destroy the PAM site and/or homology to the spacer sequence is recommended to enhance editing efficiency. Finally, testing a minimum of nine epegRNAs and three ngRNAs is recommended whenever possible (Figure 2).^4^

Potentially the most daunting hurdle to developing PE-based medicine is the jump from workhorse cell lines to primary patient cells. Nearly universally, researchers report significant losses in transfection and/or editing efficiency in primary cells. This strengthens the case for *ex vivo* therapy, in which a small proportion of successfully edited cells can be isolated from a bulk population and expanded *in vitro*. Notably, this requires a selection protocol for isolating edited cells, either via selection markers (which are generally not amenable to clinical translation) or via single-cell cloning (which is more difficult to achieve in primary cells) (Figure 2).

The advent of PASTE and PASSIGE has further revolutionized the field of prime-editing-based medicine. The possibility of integrating entire therapeutic genes into safe harbors of the genome is enticing as a permanent and mutation-agnostic treatment. Additionally, a small handful of e/pegRNA designs could theoretically be used for any disease target, with a spacer/PBS compatible with one of a few safe harbor loci, and an RT template encoding an *attP* or *attB* recombination site as needed.

The rapid pace at which PE systems are developed has yielded an increasingly branching pathway for researchers in designing PE strategies to suit their specific experimental needs. The modularity of PE architecture, multiple options for e/pegRNA design, and cell-specific delivery formats have created a wide and rich landscape for PE research.

## Acknowledgments

This work was supported by the 10.13039/501100000296British Skin Foundation (BSF) PhD Studentship Award (ST 13322), Advanced Therapies for Regenerative Medicine 10.13039/100010269Wellcome Trust PhD Training Program (218452/Z/19/Z) at King’s College London, and by 10.13039/100008081DEBRA UK (RE 22694) and 10.13039/100013786Cure EB (RE 20643). Figures were made with BioRender.

## Author contributions

I.R.B., C.G., and A.K. contributed equally to conceptualization, research, writing, and editing. J.J.-M. contributed conceptualization, editing, and supervision.

## Declaration of interests

The authors have no interests to declare.
